# Stability of Wheat Floret Metabolites during Untargeted Metabolomics Studies

**DOI:** 10.3390/metabo12010062

**Published:** 2022-01-11

**Authors:** Kristin Whitney, Gerardo Gracia-Gonzalez, Senay Simsek

**Affiliations:** 1 Department of Food Science, Purdue University, West Lafayette, IN 47907, USA; klwhitne@purdue.edu; 2 Department of Plant Science, North Dakota State University, Fargo, ND 58102, USA; miray39@yahoo.com

**Keywords:** targeted metabolomics, sample stability, liquid chromatography-mass spectrometry/quadrapol time of flight (LC-MS/QTOF), data acquisition, wheat, mycotoxins

## Abstract

A typical metabolomic analysis consists of a multi-step procedure. Variation can be introduced in any analysis segment if proper care in quality assurance is not taken, thus compromising the final results. Sample stability is one of those factors. Although sophisticated studies addressing sample decay over time have been performed in the medical field, they are emerging in plant metabolomics. Here, we focus on the stability of wheat floret extracts on queue inside an auto-injector held at 25 °C. The objective was to locate an analytical time window from extraction to injection with no significant difference occurring in the sample. Total ion current chromatograms, principal component analysis, and volcano plots were used to measure changes in the samples. Results indicate a maximum work window time of 7:45 h for Steele-ND wheat methanolic extractions in an auto-sampler at 25 °C. Comparisons showed a significant gradual increase in the number and intensity of compounds observed that may be caused by the degradation of other molecules in the sample extract. The approach can be applied as preliminary work in a metabolite profiling study, helping to set the appropriate workload to produce confident results.

## 1. Introduction

Metabolite profiling refers to the identification and quantitation of low molecular weight molecules that may be found in a particular metabolic pathway using hyphenated analytical techniques such as LC-MS/MS [1,2]. A typical metabolomic analysis consists of sample preparation, extraction, chemical analysis, data collection, data pre-treatment, data analysis, and interpretation. Variation and inconsistency can be introduced in any segment of this multi-step process; thus, it is important to give the same weight to each of its parts; otherwise, conclusions could be misleading [3,4].

One of the many analytical technologies used for the study of metabolites is liquid chromatography coupled to high-resolution mass spectrometry, or LC-HR-MS. This analytical technique has been widely used for its capacity to separate and detect a diverse set of molecules with high sensitivity [2,5]. An example of a comprehensive untargeted protocol using an ultra-high-performance LC-MS by De Vos et al. [6] resulted in the detection of several hundred metabolites from the analysis of *Arabidopsis* seedlings. In addition, this work shows quality assurance results that ensure the stability of the masses detected for 240 h [6].

Application of metabolomics in cereal science has been substantial in maize, rice, wheat, barley, oat, and rye [3,7,8,9]. Wheat has been investigated using metabolomics for a variety of phenomena. For example, ultra-performance liquid chromatography–tandem mass spectroscopy (UPLC-MS) was used to conduct integrated metabolomics–transcriptomics analysis of wheat plants infected with *Fusarium graminearum* [10]. The formation of mycotoxins in wheat has been investigated using UPLC-HR-MS [7]. LC-MS was used to conduct metabolic profiling of the genetically modified (GM) wheat T349 and non-GM cultivars to identify the metabolic basis of stress tolerance and for assessment of unexpected effects of exogenous gene insertion [11]. Metabolomics studies were also conducted to evaluate the environmental effects on genotype, free amino acids of released wheat varieties, and experimental lines [12] and compare conventional and organic farming systems over time using durum lines [13]. In another study, wheat metabolomics was applied for mapping the variation of European wheat cultivar profiles using NMR technology [14]. Ultra-high-performance liquid chromatography coupled with a time-of-flight mass spectrometry approach (UHPLC-TOF) was utilized in the comparison of diverse wheat representing durum, soft wheat, and hard bread wheat to detect desirable agronomic and human health traits [15]. Other researchers have conducted a case–control study on metabolic changes in maize in response to *Fusarium verticilliodes* infection [16].

Due to the diverse nature of metabolites, their complete recovery in a given sample has proven to be difficult. Moreover, it should also be considered how many of the metabolites are prone to decay due to events such as oxidation and hydrolysis during sample preparation [17]. An important consideration is that the goal of an extraction method should be to minimally alter the sample to avoid the increase in or degradation of metabolites [18]. A practical and common way to do this involves submerging the specimen in liquid nitrogen [6]. The rationale for this technique is to stop all enzymatic reactions and reduce the rate of chemical reactions in order to get a snapshot of the metabolites at the time of collection [19].

Shock freezing at −80 °C and freeze-drying are two recommendations that exist in sample preservation after the collection of tissue [19,20]. A few studies deal with the effects of storing the sample after extraction and before its injection into the analytical equipment [21,22]. Sophisticated studies in sample decay over time have been researched in biofluids such as urine [23,24] and blood [24,25], and also in human cell lines and mouse liver tissue [26]. The investigators studied the changes that occurred from short- or long-term cryo-preservations and from freezing and thawing the samples, providing the conditions and time best suited for sample preservation in medical metabolomics. They illustrate the importance of having quality assurance procedures integrated into the total design of an experiment.

As mentioned before, the cited studies are representative of medical research. In contrast, this approach is not often seen in plant metabolite profiling studies [27,28], specifically in wheat developing tissues analyzed in a UPLC-MS environment. There are many publications regarding the handling and storing of samples and extracted material before analysis. These studies often present very detailed methodologies but do not indicate the length of time that samples are held inside the auto-sampler [24,26,29]. Often, validation criteria will be reported for metabolomics studies. However, these reported parameters indicate the limit of detection (LOD), the limit of quantification (LOQ), precision, trueness, extraction efficiency, and matrix effects [30]. All or some of these parameters may be utilized depending on study design and if the study is untargeted or targeted [29]. Little consideration has been given to the stability of samples during analysis. Metabolomics studies are often done on a large scale that requires consideration of the duration of the run time of a sample set. Our interest in this study was not to identify the metabolites in the samples but rather to establish an analysis time window before significant changes occur during an analytical cycle.

## 2. Results and Discussion

In this study, we investigated the stability of samples as part of the initial steps in designing an untargeted metabolite profiling study. It has been recommended that samples should be analyzed as soon as extracted to prevent the decay of metabolites [31]. Often this is not possible, and measures to preserve the integrity of the extracted sample, such as reduced temperatures, darkness, enzyme addition, inert gases, etc., are commonly employed [6,31,32]. The number of samples or replicates in a single analytical run and the time for analysis need to be considered to prevent a potential change within an extracted sample that is waiting in an auto-injector. It is evident that a practical approach must be taken to ensure sample throughput as well as high recovery of metabolites [18]. Therefore, detecting the threshold of change for the type of tissue to be analyzed becomes a necessity that could be related to each plant species [33] and is also justifiable at the level of plant organs [22]. Table 1 illustrates the injection schedule. The time count began with the addition of extraction solvent to the ground floret samples until the time of their injection.

### 2.1. Extract Stability at Room Temperature (25 °C)

#### 2.1.1. Sample Reproducibility

**Figure 1:**
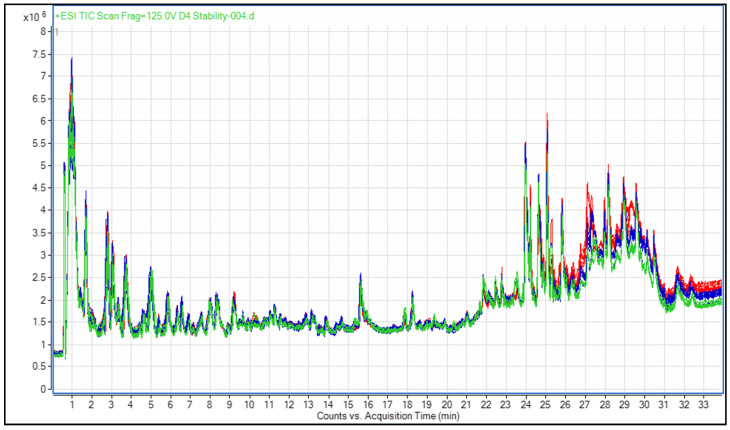
Overlaid TIC plot for Steele-ND at −80 °C analyzed on 3 consecutive days (counts per seconds vs. time in min). Each color represents a day: red = day 1; blue = day 2; green = day 3.

The reproducibility of the data acquired daily from ground floret samples stored at −80 °C was demonstrated by overlaying total ion current (TIC) plots for all runs [26,34]. Samples for day 1 (red), day 2 (blue), and day 3 (green) correspond to each day in Figure 1. Samples were injected at eight time points on each of the three days. Overall, samples appeared consistent, with a time drift of 0.1 min.

Intensities overall appear similar except from minute 25 onwards, when a slight decrease in the signals is seen as the analysis day progressed. It can be seen that samples’ signals in this experiment were stable overall. Reproducibility of retention time and intensity is important for the identification of compounds, comparison of treatments and samples, and utilization and development of compound libraries [29]. A previous study on serum samples showed a high degree of sample stability over 24 h [24]. However, in certain situations, such as equipment failure or operator error, samples may need to be reanalyzed after 24 h from the point of collection or preparation. In some cases, it may not be possible to prepare a new sample for analysis. Therefore, it is important to know the degree of variation that will be caused by the re-injection of a sample over a time period longer than 24 h.

#### 2.1.2. Data Pre-Processing

**Figure 2:**
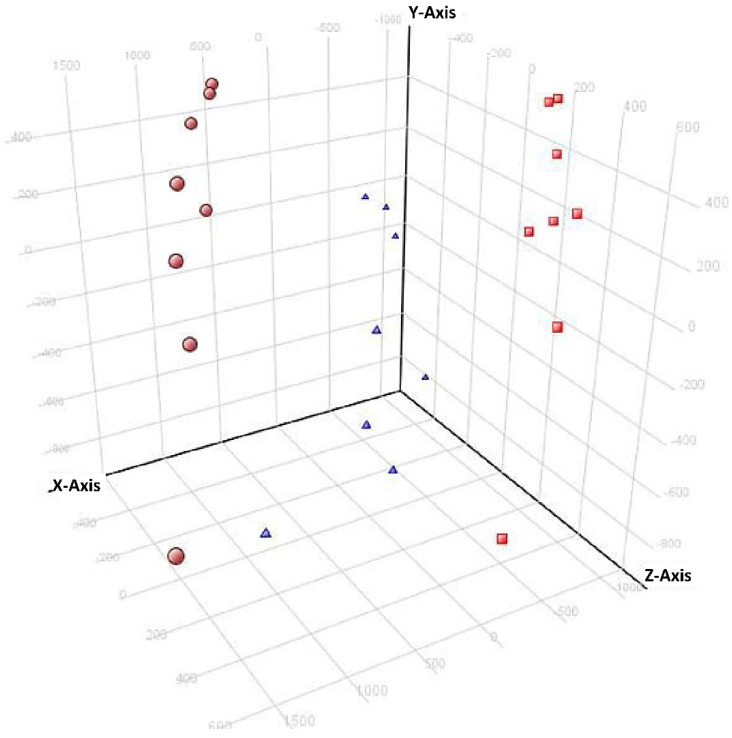
PCA of Steele-ND samples at −80 °C in 3 analytical days. The top of the Y-axis corresponds to the first injections. The most distant points correspond to the last injections. Each color represents a day: red = day 1; blue = day 2; brown = day 3. Each point represents two samples.

The blanked features served as an input for a Venn diagram, PCA, and volcano plot analyses. A total of 870 extracted features (m/z with retention times) were compiled after blanking and filtering. PCA of the ground sample stored at −80 °C was performed in order to identify any patterns. In Figure 2, storage days can be seen forming distinct clusters. Interestingly, a marked regular tendency of the time points is replicated in each day.

**Figure 3:**
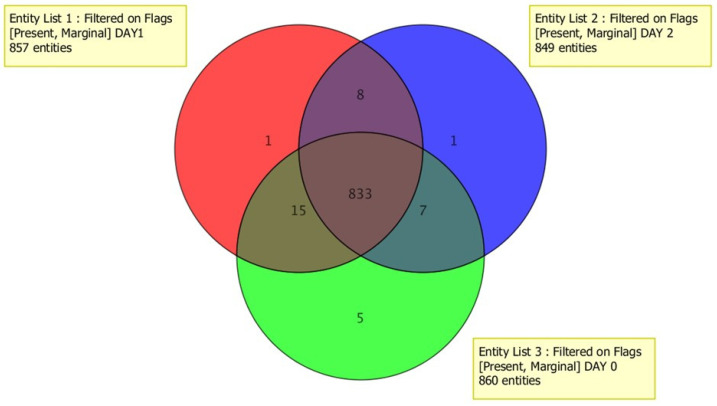
Venn diagram representing features of 3 days of analysis. Common compounds for the three days represent 95.6% of the total. Green = day 1; red = day 2; blue = day 3.

A Venn diagram (Figure 3) was used to show how many of those features were unique each day to verify the consistency of the features detected each day. The majority of features (95.6%) are common to all days, with only a few shared ones that diminished as the day progressed. The decay of features over time was evaluated by using the common pool from the three-day runs. This effectively disregarded any other molecule that entered by small differences due to preparation.

**Figure 4:**
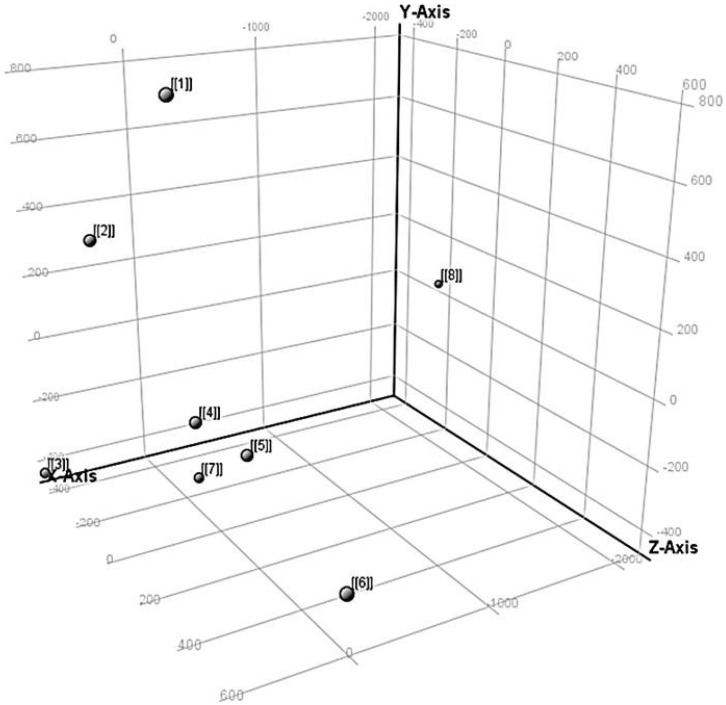
PCA score plot of Steele-ND wheat floret samples analyzed at 25 °C. Numbers 1 to 7 represent consecutive injections in the first 13 h. Point 8 represents the same sample analyzed after 26 h. Each point on the plot corresponds to 6 total samples analyzed in 3 days. Time points (hour): 1 = 5:15; 2 = 6:30; 3 = 7:45; 4 = 9:00; 5 = 10:15; 6 = 11:30; 7 = 12:45; 8 = 27:15.

Figure 4 shows the general tendency of the time points after combining the data from the three-day PCA. The most noticeable grouping is seen from 4 (9:00 h) to 7 (12:45 h). Points 1 (5:15 h), 2 (6:30 h.), and 3 (7:45 h) are far on the X-axis but are not as separated as with time point 8 (27:15 h).

### 2.2. Analytical Window Determination

**Figure 5:**
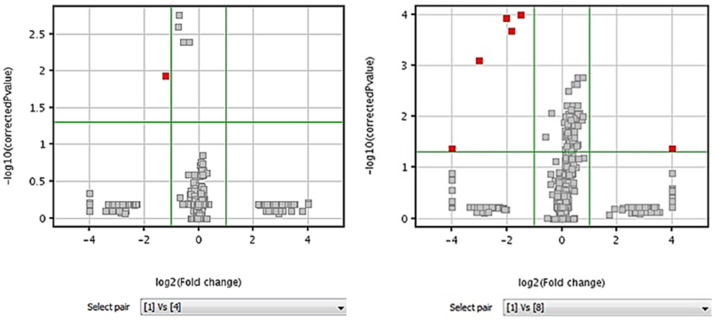
Volcano plots of Steele-ND wheat floret extracts analyzed at 25 °C. The volcano plot on the left represents time point 1 vs. time point 4, when the first significant difference was detected (9:00 h). A significant compound (242.0425 Da) was consistently found at all analyzed times. The volcano plot on the right (1 vs. 8) represents the same sample analyzed after 27:15 h. Additional significant compounds can be seen represented in red. The upper-left quadrant represents a significantly higher compound compared to any of the later time points. Features found in the upper-right quadrant represent those being higher at time point 1. No significant compounds were found in previous comparisons (1 vs. 2 or 3 time points).

A series of volcano plots comparing the initial and subsequent injections was evaluated to identify which time point was the first in which significant changes occurred to the extract. A volcano plot consists of a scatter plot in which the X-axis corresponds to the log10 of the *p*-value; the Y-axis refers to the log2 of the fold change. This arrangement allows distinguishing features with a biologically significant fold change and also a statistical significance. In this manner, it takes both the magnitude of change and variability into consideration. In this study, volcano plots were used to determine any change that could be significantly different from the original sample profile. The plots seen in Figure 5 starting from pair one vs. four show features that are statistically significant (above a horizontal green line) and important in magnitude (> or = a fold change shown by two vertical green lines).

These compounds are represented in red for each quadrant of the volcano plot. If the comparison is X vs. Y, the compounds that are higher in X will be visualized in the right quadrant. There were no significant compounds in pairs one vs. two or one vs. three (plots not shown). As time progressed, a marked increase in significant compounds was detected. Interestingly, the comparison one vs. eight also shows a feature that is higher in one than eight. This could represent a compound that decreased drastically in injection eight, thus being higher in injection one. In contrast, a report with *Brassica nigra* leaf tissue showed no appreciable effect in drift, intensity, or mass accuracy up to 240 h when using an auto-injector at 20 °C [6]. The same autosampler temperature was applied to barley metabolomics studies [35], and a lower one (10 °C) to wheat metabolite comparisons [15], although no stability data were shown.

Relevant significant features that are seen in the volcano plots were: isotopic mass 242.0402 Da/rt 4.31 min was found to be significant in pair one vs. four through eight. Features significant in pair one vs. six through eight corresponded to isotopic mass 297.0894 Da/rt 3.20 min and mass 205.0737 Da/rt 10.37 min. An additional feature in pair one vs. eight corresponds to isotopic mass 242.1055 Da/rt 10.21 min. The mass higher in time point one than time point eight corresponded to isotopic mass 598.4018 Da/rt 25.17 min. A cumulative time effect in the number and intensity of molecules is evident for the extracts in this kind of tissue; therefore, an estimated cut-off decision could be made depending on the type of study to be performed or if a specific metabolic target is at risk of being lost. In our case, due to the untargeted nature of this approach, any loss is important as it reflects a significant two-fold change for a potentially relevant metabolite.

**Figure 6:**
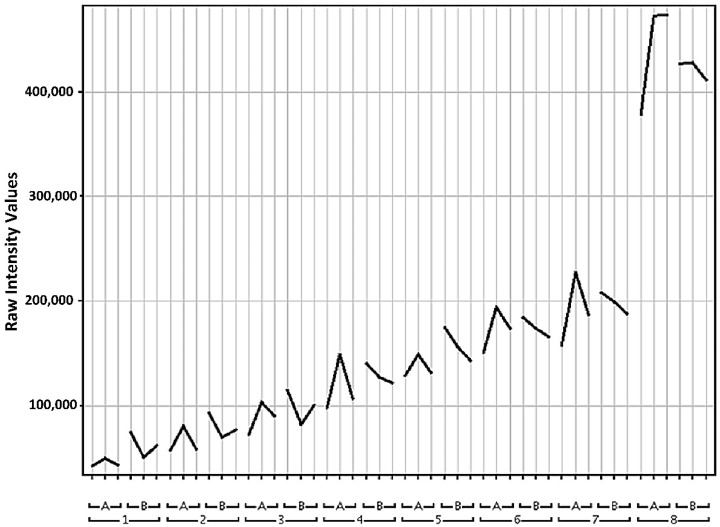
Intensity graph of a feature found in a 25 °C volcano plot 1 vs. 4 (higher in 4). The feature was common from time point 4 till 8. Intensities are plotted against each injection in succession for 3 days. Isotopic mass was 242.04025 Da with a retention time of 4.308 min. Time points (hour): 1 = 5:15; 2 = 6:30; 3 = 7:45; 4 = 9:00; 5 = 10:15; 6 = 11:34; 7 = 12:45; 8 = 27:15.

As part of their study on human biofluids, Gika, Theodoridis, and Wilson [34] verified the stability of urine samples inside an auto-injector at 4 °C. They compared the TIC of quality control samples by visually inspecting the fingerprints but relied on multivariate methods such as PCA score plots to compare the actual differences. They also found masses that increased or decreased in intensity later in the runs. The increased signals found in our study could be degradation products that intensify over time until they are detectable and determined through statistical tests. An example of one is seen in Figure 6.

For practical purposes, it was observed that for Steele-ND methanolic extracts analyzed at 25 °C, the maximum analytical time without appreciable changes should be 7:45 h. The number of samples and repeats should be fitted accordingly with this time window. This matches the concept that, aside from samples being randomly analyzed, they should be processed in reduced batches since several samples processed at a single time may increase the error [3], making it rational to make particular adjustments depending on the sample type [22,33].

As mentioned before, oxidation events are prone to occur after the sample has been altered [17]. Since samples were stored crushed in a normal air atmosphere, it makes sense that such events might happen and alter the chemistry of the samples. As a solution, crushing and extracting prior to the analysis would seem a good step to improve the overall quality at the cost of analytical time. Another improvement to prevent decay in the auto-injector could be decreasing the temperature to help preserve the quality of the sample during analysis for a longer time. Such experiments would decide the programming and repetitions of the samples to be analyzed per run cycle.

## 3. Materials and Methods

### 3.1. Wheat Planting, Inoculation, and Sampling

Seeds of the hard red spring wheat (HRSW) Steele-ND [36] were obtained from the Department of Plant Sciences of North Dakota State University (NDSU). Samples were selected based on resistance to Fusarium head blight [37]. Steele-ND’s pedigree consists of: “Parshall” (PI 613587)/5/“Grandin” (PI 531005)/3/IAS[20.sup.*]4/H567.71//“Amidon” (PI 527682)/4/[Grandin.sup.*]2/“Glupro” (PI 592759) [36].

Wheat was sown in 3.8 cm Ray Leach Cone-Tainers (RL cones), planting one seed in each cone. Sunshine Mix #1/LC1 soil was used for planting. The plant number was set at 70 cones per rack. After emergence, eight beads of Multicoat 4 fertilizer (Haifa Group, Israel) were applied to the topsoil of each cone. The racks containing the cones were kept in trays full of reverse osmosis water. Greenhouse temperature was set at 25 ± 2 °C with a 16:8 h photoperiod until the booting stage [38].

A *Fusarium graminearum* strain isolated at Foster, North Dakota in 2008, named Fg08-001, was provided by Dr. Shaobin Zhong of the Plant Pathology Department at NDSU. It is regarded as a strain with a 3-Acetyl-DON chemotype [39]. The strain was cultured in mung bean agar (40 g of mung beans per liter [wt/vol] in Milli-Q water, boiled for 23 min, and filtered through four layers of cheesecloth; 1.5% agar [wt/vol]) and was incubated at room temperature with 12 h fluorescent light cycles. After seven days, the cultures were inundated with sterile water, and the spores/mycelia were dislodged with a sterile loop. The resulting suspension was filtered through two layers of cheesecloth. A quantified spore suspension was prepared by counting macroconidia using a hemocytometer. Appropriate adjustments were performed using an aqueous solution of 0.02% Tween 80 to reach a concentration of 10^5^ macroconidia/mL [39]. Aliquots of this suspension were stored at −20 °C for later use. Prior to usage, the frozen macroconidial suspension was thawed for approximately 8 h at 4 °C. The suspension was utilized for the next three days and stored at 4 °C [38].

When the first awns were visible (GS = 47–49, Zadoks scale [40]), wheat plants were incubated in a growth room with conditions similar to the greenhouse, with the exception that the light intensity was ≈15,000 lumen/m^2^. The purpose of this was to stabilize the plants by having a constant light intensity while avoiding temperature changes. Then, at anthesis (GS = 60–69 Zadoks scale [40]), wheat florets were inoculated with *F. graminearum* spores using 10 μL of the macroconidial suspension (100 conidia per μL) injected between the palea and lemma of four central spikelets within a spike. A total of four spikes were inoculated. Inoculated spikelets were marked by cutting their respective awns. All treated plants were incubated in a mist chamber for 24 h. Mist chamber conditions were maintained at 28.5 ± 0.5 °C, with a spraying rate of 20 s at 15 min intervals to ensure 90 to 95% RH for fungal colonization [38].

After incubation, inoculated florets in each wheat spike were collected by cutting the rachilla at the base of each spikelet. A composite sample was composed of 16 pooled florets originating from four spikes. Four florets were inoculated in each spike [41]. Florets were collected in labeled #1 Kraft paper coin envelopes (2.25 by 3.50 inch). The envelopes were stapled and submerged immediately in liquid nitrogen. Frozen tissue was stored in an ultra-freezer at −80 ± 1 °C until use [38].

### 3.2. Sample Extraction

Mortars and pestles were washed with 2% phosphate-free detergent followed by a two-day distilled water soaking/rinse cycles. After drying, the ceramic was baked at 590 °C for 4 h. The frozen tissue was crushed into powder by utilizing a clean ceramic pestle and mortar sitting on dry ice. Tissue thawing was avoided by carefully pouring a small volume of liquid nitrogen onto the sample during crushing. Six composite samples were ground together, and the powder was collected into a single plastic conical tube. The tube was kept at −80 °C. Two portions of the ground tissue were weighed (marked A and B) and extracted each day of analysis, repeating the procedure for a total of 3 consecutive days [38].

Samples prepared for small molecule analysis were extracted based on the methods developed by De Vos, Moco, Lommen, Keurentjes, Bino, and Hall [6] and t’Kindt, Morreel, Deforce, Boerjan, and Van Bocxlaer [27] with modifications. Tissue extracts were prepared freshly at the beginning of each day of analysis. Extraction time was recorded as the start time and counted as part of the total analytical run. The ground, 300 mg ± 5% sample was transferred into a pre-frozen 1.5 mL microtube. Cryogenic conditions were kept by submerging the tube and spatula into liquid nitrogen. Sample extraction solution (80:20 methanol/water HPLC grade) stored at −80 °C was added in a 3:1 volume/fresh weight ratio.

Tubes were stirred in two second pulses using a vortex shaker for a total of 10 s. Next, to facilitate extraction, each sample was submerged in a Branson 2510 bath sonicator (Branson Ultrasonics Corp., Brookfield, CT, USA) at 25 °C for 15 min set on maximum frequency (40 kHz). This was followed by two sequential centrifuge cycles of 10 min each at 3000 g at 6 ± 1 °C. The supernatant of each sample was filtered through a 0.2 μm PTFE membrane (VWR International) by using 1 mL disposable syringes into new 1.8 mL amber glass vials with Teflon caps. Ambar vials labeled as blank were prepared in the same way as the samples, except they only contained extraction solution [38].

### 3.3. Sample Analysis

Metabolite data were obtained using a 6540 series UHPLC-ESI-QTOF/MS (Agilent Technologies, Inc., Santa Clara, CA, USA) utilizing the MassHunter Workstation software LC/MS Acquisition for 6200 series TOF/6500 QTOF version B.05.00/build 5.05.042.0. The Infinity 1290 UHPLC section (Agilent Technologies, Inc.) was composed of a G44227A flex cube, G4220A binary pump, G1210B Iso pump, G1316C TCL, and a G4226A sampler unit. A Zorba X Eclipse plus C-18 column (1.8 μm; 2.1 × 100 mm) was utilized. Reverse phase conditions were maintained at 40 °C with a flow rate of 0.4 mL/min. The mobile phase consisted of water with 0.1% of formic acid (solvent A) and acetonitrile with 0.1% of formic acid (solvent B) [38].

For the analysis of Steele-ND, a step gradient elution profile was used starting with 5% B for 0.75 min; increasing from 5% to 35% B between 0.75 and 15 min; from 35 to 100% B between 15 and 30 min; back from 100% to 5% B until minute 34.01. Post-run time was set at 2 min to clean the column. The injection volume was 2 μL. A blank sample was run two times before the beginning of a series of injections and at the end of the run. A needle wash of 3 s with needle seat black flush was included [38].

The UHD Accurate-Mass MS general acquisition settings and MS-TOF settings were left as default except for the parameters described in the Supplemental Material (TOF/Q-TOF Mass Spectrometer section). A reference mass solution was prepared using an API-TOF Reference Mass Solution Kit (Supelco/Agilent Part No. G1969-85001). It consisted of 1.0 mL ammonium trifluoroacetate (100 μM); 2.0 mL purine (10 μM); 0.8 mL (2.5 μM) hexakis (1H, 1H, 3H-tetrafluoropropoxy) phosphazine; dissolved in 500 mL of 95% acetonitrile: 5% water. Reference masses were 121.050873 and 922.009798 [38].

Sample vials that contained either a blank or inoculated floret extracts were held in the Infinity 1290 UHPLC auto-injector at 25 °C for the duration of the analysis run (more than 24 h.). The process was as follows: the ground Steele-ND sample was divided into two vials (A and B) and extracted. Time count started at the moment the solvent was added to the ground florets. After extraction, 7 consecutive time points were analyzed: 5:14, 6:30, 7:46, 9:02, 10:18, 11:34, and 12:50 h. After a pause, the last time point was collected at 26:29 h (Table 1). The process was repeated on three consecutive days; each one began with a fresh sample extraction. The results files of the analysis had a *.d extension [38].

### 3.4. Data Pre-Processing

Raw *.d files from the MassHunter Acquisition software were processed using MassHunter Workstation Qualitative (MH Qual) analysis ver. B.5.00 Build 5.0.519.0 (Agilent Technologies, Inc.). Within this software, the Molecular Feature Extraction (MFE) algorithm provided a *naïve* finder that was effective as a first way to scrutinize UHPLC-QTOF/MS raw data. The algorithm was set accordingly for small molecule discovery. The adjusted parameters can be found in the Supplemental Material (Find by Molecular Feature). Pre-processing was assisted by the DA re-processor software ver. B.05.00 (Agilent Technologies, Inc.), which converted *.d files to *.cef files [38].

#### 3.4.1. Mass and Time Alignments

The retention time (RT) window was estimated by the MH Qual software by overlaying the raw peaks or the total ion current (TIC) scan within each day and clicking the apex of the left-most peak of a zoomed-in plot. This time was recorded and subtracted from the right-most peak of another TIC. Comparative sample plots were assembled at this stage. The output “.cef” files were aligned for time and mass using Mass Profiler Professional (MPP) ver. 12.1 Build 170166 (Agilent Technologies, Inc.). The compound alignment option was modified to set a retention time window of 0.15 min. A filter by flags option was selected at 10% of the total samples in order to discard some of the artifacts generated by the previous processes. Flags are attributes that denote feature quality. Selected options can be: “present” (mass was detected), “absent” (no mass detected), and “marginal” (signal saturated). The filter finds masses based on the quality of these flags in the sample files. A recommended setting involves detecting the present and marginal features found in at least 2 out of 20 or fewer sample files. The features that pass the filter are collected in a new list for further processes. The masses that are only found once in the entire data file are likely to be artifacts and are discarded. Due to the number of files (more than 20), 10% of the total sample files seemed to work well, according to the manufacturer. The resulting aligned data were exported for recursion analysis using a single *.cef file [38].

#### 3.4.2. Recursion Analysis

This step was performed in the MH Qual program after completing the MFE run, mass, and time alignment. Utilizing this approach significantly reduced the false positive features generated by the MFE. The algorithm “find by formula” included: options, chromatograms, and mass spectra. The unique *.cef file generated by the alignment process was used as a template to start the recursion analysis. The specific settings can be found in the Supplementary Material (Find Compounds by Formula section). The MH Qual software was again assisted by the DA re-processor program. After the recursion analysis, the new output sample *.cef files were loaded into the MPP software for statistical analyses [38].

### 3.5. Statistical Analyses

#### 3.5.1. Blank Subtraction and Venn Diagrams

These operations were necessary to effectively subtract the features (also called entities, unidentified compounds with a mass and retention time) found in the blanks from the actual samples. It was performed using MPP software. As recommended by the manufacturer, data were log2 transformed and then filtered by flags at 10% before creating a Venn diagram to separate and save the features unique to the experimental samples. After collecting a blanked data set, another Venn diagram was prepared to obtain a common set of features between days and samples. This blanked common feature set was saved as an entity list to be used in unsupervised PCA plots to verify general tendencies and volcano plots (*t*-test) to identify statistical significance and magnitude changes throughout injection times [38].

#### 3.5.2. Interpretations

MPP software needs to create “interpretations” to answer a particular question. In this study, a “day” interpretation was created to visualize the relation between the three days. As the aim was to assess a total universe of compounds and a maximum analytical time window, we created the “injection” interpretation, which focused on time points considering the day and replicates. Other interpretations were created in this fashion to perform blanking or make other preliminary comparisons [38].

#### 3.5.3. Principal Component Analysis

All plots on experimental conditions were displayed as 3D PCA score plots on the MPP platform. The day and injection interpretations were used. The samples were analyzed as injection numbers. The PCA was carried out after filtering by flags at 10% [38].

#### 3.5.4. Volcano Plots

Since our ultimate goal was to have a practical window time for analysis, data were compiled into volcano plots for comparison. This statistical test was very useful for visualizing differences between the first and any other sample time point. It was performed using the MPP software. This arrangement allows distinguishing masses with a fold change (magnitude of change) of biological significance and also statistical significance (takes both magnitudes of change and variability into consideration). A two-fold change in magnitude represented a relevant biological change and was equal to the absolute ratio between the normalized average intensities of condition 1/condition 2. The paired *t*-test option was selected to compare the initial time point against the rest. Benjamini–Hochberg FDR multiple testing correction was added for *p*-value correction. The *p*-value cut-off was set to 0.05. The fold change cut-off was set at 2. The abundance difference (raw, abs) cut-off was set to lower the number of false-positive features originated by the process. A value of 500 was enough to eliminate meaningless fold change differences [38].

## 4. Conclusions

The effect of time on the stability of extracted wheat floret samples was investigated over a period of time during analysis. The use of PCA coupled with volcano plots helped to define the maximum time before the sample started to change in an auto-sampler. For Steele-ND wheat floret extracts analyzed in a 25 °C environment, a maximum stable analytical time (from extraction to injection) was found to be 7:45 h. The time found in this study should not be taken for any other type of sample or analytical pipeline. However, the approach could be applied to determine the work logistics in a metabolite profiling study with the aim of producing confident results. Overall, this study provides some clarification regarding the stability of compounds in a crude methanolic extract at room temperature (25 °C) for metabolomic profiling of wheat florets. Going forward, it will be beneficial to expand the scope of this work based on other solvent extract types and other sample holding temperatures. We would recommend determining sample stability in other common extraction solvents and for other extraction methods. Additionally, we recommend determining sample stability at different temperatures, such as 4 °C or 30 °C, to ensure the stability of compounds of interest in various analysis conditions.

## Figures and Tables

**Table 1:** Cumulative time sequence for injections at 25 °C.

Time Point	Injection Time (h:min) ^a^
1	5:15
2	6:30
3	7:45
4	9:00
5	10:15
6	11:30
7	12:45
8	27:15

^a^ Time in hours and minutes taken for each time point over three consecutive days.

## Data Availability

No new data were created or analyzed in this study. Data sharing is not applicable to this article.
